# Can Syphilis Be Cured with High Potencies?

**Published:** 1898-07

**Authors:** J. M. Selfridge

**Affiliations:** Oakland, Cal.


### CAN SYPHILIS BE CURED WITH HIGH POTENCIES?

J. M. Selfridge, M. D., Oakland, Cal.

Mr. President:—Before attempting to answer this question it will be of interest to inquire as to the nature and origin of syphilis.

It is generally understood to mean a sore on some portion of the genitalia, called a chancre; but this sore and its sequences are not the disease, but rather the results of diseased action.

Hahnemann defines disease as disturbed vital force. But what, in this case, is the disturber of the vital equilibrium—or, to speak more correctly, what is it that disturbs the normal molecular activities in such a manner as to produce a disease so far-reaching, so terrible in its sequences, and so unlike every other disease?

Much has been said and written on this subject by eminent authors, but no one seems to have been able to solve this knotty question. I know, of course, that bacteriologists claim that it is a specific germ, and, although there are those who claim to have discovered such a germ, still the fact remains that no one has been able to isolate an organism that has been accepted by all pathologists as the true bacillus syphilitica. While this is undoubtedly true, it is admitted by all authorities that there is a specific poison with which the system becomes inoculated during sexual intercourse. But, as Ericksen says, "the exact nature of the virus is unknown." (Edition 1895.)

It is said to have been epidemic in the fifteenth century, and its results have been so terrible that no nation or people has been willing to acknowledge that it originated with them. Hence we find the Italians, English, and Germans calling it the French disease, while the French lay it to the Italians, and the Spanish claim it was brought from America by the followers of Columbus. Whether it originated in China or elsewhere, there is good authority for stating that it existed in the "Flowery Kingdom" twenty-five centuries before the Christian era.

But, to my mind, it is unfair to accuse one nation or one class of people more than another, when the truth lies in a nut-shell; too frequent and too promiscuous sexual intercourse, coupled with filth and intoxication, will produce it among any people, nation, or city. Moral purity and cleanliness were never visited by either syphilis, chancroid, or gonorrhœa.

Now as to the *treatment*. Ever since the latter part of the fifteenth century Mercury, in one form or another, has been considered the specific remedy for this disease. Agnew recommends the bi-chloride of Mercury, the one-twentieth of a grain after meals, to be continued not less than eighteen months.

Moulin says the administration should be quietly but steadily increased until the teeth begin to feel loose. He recommends Hydrarg. Cum Creta in two-grain doses three times a day; while Ericksen gives the "gray powder" coupled with Opium, when necessary, to restrain the bowels.

E. L. Keys prefers the proto-iodide of Mercury. He commences with the one-sixth of a grain three times a day, and after three days he gradually increases it by adding from one to two granules daily until the maximum dose is reached, which declares itself by a watery diarrhœa or by touching the gums. Duration of administration from eighteen months to two and one-half years, and in some cases four years. The size of the full dose varies greatly for different individuals, and is invariably given after meals. This treatment, with some modifications, is recommended by Morrow and Horwitz. But, no matter when or by whom recommended, the objective point is the same—that is, to bring the system thoroughly under the influence of Mercury, but, if possible, without producing salivation.

Under certain circumstances these modern authors recommend a mixed treatment of the bi-chloride of Mercury and the iodide of Potassium. Such a mixture reminds a person of the prayer of a certain sinner who thought he was about to die. He called upon "Good Lord," "Good devil," because he did not know into whose hands he might fall.

When secondary symptoms show themselves the iodide of Potassium is recommended in doses ranging from ten or twenty grains three times a day, to be increased, in some cases, to two and one-half ounces daily.

To a physician well versed in the teachings of Hahnemann such dosing is simply appalling. These modern broken doses of Mercury, although they would have been very unorthodox fifty years ago, yet, when continued from eighteen months to two and one-half years, would fill a patient sufficiently full of Mercury to make him a walking barometer were it not that nature is so arranged that a portion of the drug is eliminated. The allopaths claim that it is all removed from the system in time; but, from experience, we know that a certain amount of it becomes as fixed in the liver and elsewhere as the India ink does after being tatooed. By this same eliminating process a portion of the iodide of Potassium is removed. Were this not the case, a sufficient quantity of the alkali would accumulate in the system to convert the adipose tissue into soap. Were such the fact, what a spectacle some of these fat fellows would make! If we were to assume that this method of treatment were the correct one, it is certainly not scientific to introduce more of the remedy into the system than is required to cure. That they, the allopaths, do err in this direction, is evident from the fact that nature cannot appropriate all of the remedy, and, therefore, a portion of it is eliminated by the emunctories.

Seventy-five or more years ago Hahnemann taught what the more advanced allopaths are beginning to recognize as true, viz., that it is bad practice to destroy the chancre by cauterization or any other external application, because the healing of the chancre does not cure the constitutional malady. While condemning the methods in use during his time Hahnemann makes the somewhat startling statement that syphilis is as easily and promptly cured as any other disease.

In outlining his method he uses the following language: "In the cure of venereal disease three states are to be distinguished:

"First—When syphilis is still alone and attended with its local symptom, the chancre, or at least, if this has been removed by external applications, it is still associated with the other local symptom—the bubo.

"Second—When it is alone—*i. e.*, without any complication with a second or third miasm, but has been deprived of the vicarious local symptom, the chancre (or bubo), it may be cured the same as the first, but the certainty of a cure can not be so easily determined, because the chancre has been removed by external means.

"Third—When it is already complicated with another chronic miasm—*i. e.*, with psora already developed, while the local symptom may either be present, or may have been removed by local applications."

He further says that "The most difficult of all these cases, the *third*, is when a man, at the time of the syphilitic infection, was already laboring under a chronic disease, so that his syphilis was complicated with psora," or when he has been weakened by allopathic dosing so that his general health has been undermined, "the chancre having been destroyed by local means. When so complicated with developed psora it cannot be cured without curing the psora also." In speaking of the remedy and the method of administration Hahnemann is concise and positive. He says, "When syphilis is uncomplicated with psora, when the chancre (or bubo) is still present—in the first state, it needs only one little dose of the best mercurial remedy in order to cure thoroughly and forever the whole syphilis with its chancre within fourteen days."

The "mercurial remedy" which he recommends is the thirtieth centesimal potency of Mercurius-vivus. Is it possible that one small dose of the thirtieth potency of Mercurius-vivus will cure a loathsome disease like syphilis in fourteen days when it takes the allopaths two and one-half years, with loads of medicine? And then they are not sure that the patient is cured, for they have to stand guard another year, when, if no untoward symptoms make their appearance, they venture to guess that their patients are cured. But are they cured? Clinical experience would seem to give a negative answer.

Hahnemann has said, When there are no psoric complications that the single dose will cure. Can it be that so careful an observer as he has proved himself to be would risk the health and lives of thousands of human beings, to say nothing of the risk of his own world-wide reputation—of the risk to that system of medicine that had cost him so many years of hard study, that system of medicine that was dearer to him than life itself—if he did not know whereof he spoke? The thought of such a thing is simply absurd.

Does any person doubt Hahnemann's honesty? Paralyzed be the tongue of him who would *dare* to express such a sentiment! And yet there are those who profess to believe the law of similars, who express a doubt in *acts,* if not in words. Why do they doubt? For the simple reason that they are either ignorant of what he teaches, or they have not the courage to put his teachings into practice. They prefer to follow the unscientific methods of the dominant school.

In addition to this Hahnemann tells us how we may know when a case of syphilis is cured. He says: "In case the chancre has been driven out through local applications, even if the remedies had not been very acrid, there will always remain in the place where it stood, as a sign of unextinguished internal syphilis a discolored, reddish, red, or blue scar, while, on the contrary, when the cure of the whole venereal disease has been effected by the internal remedy, and the chancre heals of itself without the action of an external application, then the spot of the former chancre can be no more recognized, for the skin covering that place will be just as smooth and of the same color as the rest."

This being true, we do not have to wait a year after the treatment is stopped before pronouncing a case of syphilis cured.

This being true, how much more simple, how much more certain, how much more safe, and, therefore, how much better for the patient is *pure Homœopathy* than the "flesh pots of Egypt," than the risk of two and one-half years' drugging of Keyes, Molin, and others. I, therefore, in the language of Joshua of old (slightly paraphrased) call upon all of you who profess to be homœopaths, to choose ye this day whom ye will serve, whether God or Baal, but as for me and all whom I can influence, we will follow *Hahnemann and pure Homœopathy* rather than *Keyes and Allopathy*.

But, Mr. President, this paper will not be complete without the following report of a case:

December 15th, 1896, P. W.——, aged sixteen years, came to my office to be treated for an injury which he said he had received to his penis. In response to questions he said it was sore, red, and swollen. Being in a hurry at that moment, I did not ask to inspect the member, but prescribed Arnica, to be taken internally, and instructed him to bathe the part three or four times a day with water. I heard nothing from him until January 4th, 1897, when he returned to say he was worse—that the glands in his groin were swollen. I then asked to see the organ, and, to my surprise, I found the young man had lied to me on his previous visit. His Ebenezer was swollen to more than twice its normal size. There was phymosis and some discharge from the prepuce. The upper right side of the glans at the coronal margin was more tender to touch than the surrounding parts, and there were two buboes in his right groin above Poupart's ligament. I scolded him roundly for deceiving me, and, after closely questioning him, he admitted having had sexual intercourse with a prostitute about six weeks previous; that when he first visited me there were at that time two sores at the point above described, which made their appearance about a month after he had been with this person. The lad had syphilis, and, because of the phymosis, I could not see the chancres. I did not circumcise him, or slit up the foreskin, but, as the case had over two weeks the start of me, I gave him Mercurius-vivus 30 (B. & T.), a dose every four hours, with instructions to syringe the prepuce three times a day with water as hot as he could bear it.

January 16th, as he was no worse, I gave S. L.

January 28th, as there was no improvement, I prescribed ℞—Merc-viv. 30th.

February 7th, ℞—Merc-viv. 30th.

February 18th, the swelling and soreness of the penis had about subsided, and the discharge had ceased. ℞—S. L.

March 2d, his hair was falling out. Syphilides were making their appearance on his thighs, and there were mucous patches on his fauces. ℞—Merc-viv. 30 every four hours.

March 16th, syphilides were quite plentiful over the entire body, and his hair had dropped out in patches so that it was more than half gone. ℞—Syphilinum CM. one dose.

March 27th, there was no improvement. ℞—Syphilinum CM. one dose.

April 26th, one month after the second dose of Syphilinum, he was very much improved. His hair had not only ceased to fall, but in the bald spots a new growth of hair was showing itself, and the mucous patches were decreasing in size.

It is my custom, when a patient is improving, to either stop the remedy or lengthen the interval of its administration, but in this case I gave him one more dose of Syphilinum CM.

May 7th, the syphilides had entirely disappeared. A new crop of hair was very perceptible, and the preputial orifice had so far recovered its normal elasticity that he could uncover about one-half of the glans. But, as the buboes had not disappeared, I prescribed Mercurius-viv. 30, two powders to be taken mornings.

May 30th, I gave two more powders to be taken the same as before. I did not see him again until

November 15th, when he appeared to be perfectly well. There was not a trace of the buboes, mucous patches, or syphilides. He could uncover the glans with perfect ease, and there, side by side, were two places where the chancres had been, but, except for a slight depression, there was nothing to distinguish them from the rest of the glans. The surface was smooth and the color normal.

On the 2d of February, 1898, he came to me with a growth on his under lip which had every appearance of an ordinary wart. As the base was broad and fleshy I prescribed Causticum 200, to be taken mornings. At the end of three weeks it had entirely disappeared.

Was the wart syphilitic? I think not. In my opinion, it was of psoric origin. But the psora in this case must have been latent, for, had it been well developed when the patient contracted syphilis, I would have had to cure the psora before the syphilis would have yielded to an indicated remedy. But, the psora being latent, the wart did not make its appearance until after the syphilis was cured.

RESUMÉ.

The treatment for the syphilis was commenced January 4th, 1897.

March 2d his hair was falling out rapidly, and syphilides were making their appearance, and as they were increasing under the use of Mercury, three doses of Syphilinum CM. were administered between the 16th of March and 7th of May, when the syphilides had entirely disappeared.

Mercury was then resumed, because the buboes had not disappeared, but he took no medicine after the 2d of June.

On the 15th of November every trace of the disease, as before stated, had entirely disappeared.

The patient took medicine at intervals just five months, during which time the largest dose administered was the 30th potency of Mercurius-vivus, and the smallest dose was the one-hundred thousandth of Syphilinum.

It may be asked, Were these prescriptions purely Hahnemannian? I answer, yes and no. Yes, as to the remedy and potency of Mercurius, but no as to the repetition of the dose.

It may be said that the repetition of the dose could have made no difference with the result. But, Hahnemann, who was a better observer than either of us, has said that it does. In his directions for proving drugs, in Section 131 of *The Organon*, he says, "and besides a second dose, by its curative effect, will often remove some of the symptoms resulting from the previous dose; or a second dose may produce the oppposite condition from that of the first."

Let me say, in passing, that it is quite probable that this suggestion of Hahnemann gave Bœnninghausen the idea that a high potency will antidote the effects of a lower potency of the same remedy—a discovery which has since been more fully elaborated by Dr. Sawyer, of Chicago.

Again, in Section 245, Hahnemann says: "Perceptible and continued progress of improvement in an acute or chronic disease is a condition which, as long as it lasts, invariably counter-indicates the repetition of any medicine whatever, because the beneficial effect which the medicine continues to exert is rapidly approaching perfection. Under these circumstances every new dose of medicine, even of the last one that proved beneficial, would disturb the process of recovery."

And again, in Section 247, he says that "Homœopathic medicines may be repeated with excellent and often astonishing effect, at intervals of fourteen, twelve, ten, eight, or seven days." But we must not infer from this quotation that Hahnemann means that this shall be the rule in all diseases, for further on in *The Organon* he directs that, in diseases whose progress is rapid, like cholera, for example, the remedy may be repeated every five minutes.

But in syphilis his experience, as before stated, is one dose and a cure in fourteen days.

Had I followed his teaching in the case here reported it is altogether probable that no syphilides would have made their appearance; but my anxiety to make a prompt impression on so bad a case led me to forget the precepts of the master, and made it necessary for me to resort to another remedy, which, although it did splendid work, its use might have been avoided.

Were it necessary to prove the truth of Hahnemann's statements, other cases might be mentioned, but this is sufficient to establish the fact that syphilis *can* be cured with *high potencies.*

---

[In reference to Dr. Selfridge's remarks concerning the origin of syphilis the reader who wishes complete information on the subject should read Dr. F. Buret's work, entitled *Syphilis in Ancient and Prehistoric Times*, translated by A. H. Ohmann-Dumesnil, M. D. This author shows that syphilis is as old as man. A review of the first volume was written by the Editor of this journal and published in The Homœopathic Physician for September, 1892. The second volume was similarly reviewed in this journal for June, 1896. These two reviews give an epitome of the whole book, and might be useful to peruse by those who have not the time to read the whole book.

The Editor has had a late experience in curing syphilis completely in three months with high potencies, thus corroborating Dr. Selfridge's statements in the foregoing admirable article to which this note is appended.—Ed.]

